# Isolation and characterization of an antifungal compound 5-hydroxy-7,4’-dimethoxyflavone from *Combretum zeyheri*

**DOI:** 10.1186/s12906-015-0934-7

**Published:** 2015-11-14

**Authors:** Rumbidzai Mangoyi, Jacob Midiwo, Stanley Mukanganyama

**Affiliations:** Department of Biochemistry, University of Zimbabwe, P.O. Box MP 167, Harare, Mt. Pleasant Zimbabwe; Department of Chemistry, University of Nairobi, Nairobi, Kenya

**Keywords:** *Combretum zeyheri*, Antifungal, 5-hydroxy-7, 4’-dimethoxyflavone, Chromatography, Isolation

## Abstract

**Background:**

*Combretum zeyheri*, belongs to the family *Combretaceae* and is one of the most popular herbal plants in tropical and subtropical countries. The leaves of *Combretum zeyheri* have been used as herbal medicine and have been reported to have pharmacological activity which includes anti-bacterial, anti-fungal, anticancer and antioxidant properties. The goal of this study was to isolate, identify and characterize compounds from *C. zeyheri* leaves which are responsible for its antifungal activity.

**Methods:**

The preliminary isolation of *C. zeyheri* active compounds was carried out using chromatographic techniques which include sephadex gel column chromatography, silica gel column chromatography and thin-layer chromatography (TLC). The isolated compounds were then investigated for their antifungal activity using broth dilution assay. The combined effect of the most potent compound and an antifungal drug miconazole was investigated using the checkerboard assay. Time-kill assays were conducted for the combinations using the colony counting method. The mechanism of action of 5-hydroxy-7,4’-dimethoxyflavone as a potent antifungal agent was investigated by determining its inhibitory activity on *Candida albicans* drug efflux pumps using the ciprofloxacin assay. The ability of 5-hydroxy-7,4’-dimethoxyflavone to inhibit antioxidant enzymes as well as the biosynthesis of ergosterol were also investigated.

**Results:**

A total of four pure compounds (A-D) were isolated from *C. zeyheri* leaf extract. Compound B (5-hydroxy-7,4’-dimethoxyflavone) was found to be active against *Candida albicans* using broth dilution method. This compound was also found to have synergistic activity on growth of *C. albicans* when combined with miconazole, completely inhibiting growth after only 4 hrs of incubation. Analysis of ergosterol content from *Candida albicans* showed a time-dependent decrease to 91 % and 63 % at 16 and 24 hrs respectively, in cells treated with ½ MIC of 5-hydroxy-7,4’-dimethoxyflavone. The compound 5-hydroxy-7,4’-dimethoxyflavone also showed inhibition of both the drug efflux pumps (with IC_50_ = 51.64 μg/ml) and the antioxidant enzymes (at 5 μM).

**Conclusion:**

The compound 5-hydroxy-7,4’-dimethoxyflavone may be partly responsible for the reported antifungal activity of *C. zeyheri*, and may serve as a potential source of lead compounds that can be developed as antifungal phytomedicines.

## Background

Since 1970s, the emergence of antifungal drug resistance has been a cause of concern in the treatment of invasive fungal infections such as candidiasis [1]. This is particularly due to increased use of triazoles in prophylactic and empiric antifungal therapy in high-risk patients [2]. Persons who are at high risk for systemic fungal infections include those who have haematological malignancies, undergoing prolonged bone marrow transplantation and chemotherapy, HIV infected patients and severely burnt patients [3]. Although many of the antifungal drugs have advanced the management of fungal infections, failure rates remain high as the discovery of the ideal antifungal agent against resistant strains has not been yet obtained [4, 5]. Thus, there is need for better strategies to protect existing drugs and develop new classes of effective drugs to treat resistant infections.

Since 1981, researchers have paid attention to the potential of medicinal plants as alternative sources for the isolation of novel metabolites with interesting biological and pharmaceutical properties [6]. Medicinal plants have been used extensively as crude material or as pure compounds for treating various disease conditions. The World Health Organization has estimated that almost 65 % of the world’s inhabitants rely mainly on traditional medicines for their primary health care [7]. This is because traditional medicine has remained as the most affordable and easily accessible source of treatment in the primary healthcare system mainly in most developing countries [8].

In Africa, *Combretum* species are well known in traditional medicine and used for various ailments and diseases ranging from heart and worm remedies to wound dressings, treatment of the mentally ill, scorpion stings and snake bites, to fever and microbial infections [9]. Among antimicrobially active compounds isolated from *Combretum* species are combretastatins (bibenzyle compounds), acidic tetracyclic and pentacyclic triterpenes, ellagitannins, phenanthrenes, flavonoids, saponins and cycloartane glycosides [10].

We previously reported that *Combretum zeyheri* had antifungal activity against *C. albicans* and *C. krusei* [11]. It showed growth inhibitory activity against *C. albicans* and *C. krusei* with minimum inhibitory concentrations of 0.08 and 0.16 mg/ml respectively using the broth dilution method [10]. Partial bioguided fractionation of *C. zeyheri* extract by sephadex LH20 gel column chromatography enabled isolation of the fractions which were also found to have the antifungal activity as well as the drug efflux pumps inhibitory activity [12].

*Combretum zeyheri* has also been reported to have antimicrobial [10] and antitumor activity [13] as well as the antioxidant and anti-inflammatory activity [14]. Thus, these previous findings show that *C. zeyheri* plant has great antimicrobial potential and may be used as a source of lead compounds for the development of antimicrobial drugs. Therefore, the objective of this study was to isolate the active antifungal compounds from the crude ethanolic leaf extract of *C. zeyheri* using chromatographic techniques and then investigate the mechanism of action of these active compounds.

## Methods

### Fungi and reagents

Reserpine, rhodamine 6G, nutrient agar, sabouraud dextrose agar (SDA), miconazole, dimethyl sulphoxide (DMSO), silica gel 60 – 120 mesh, 3-(4,5-dimethylthiazol-2-yl)-2,5-diphenyltetrazolium bromide (MTT), phenylmethylsulphonyle fluoride (PMSF), sucrose, 1-chloro-2,4-dinitrobenzene (CDNB), glutathione, hydrogen peroxide (H_2_O_2_), epinephrine, adrenochrome, NADPH, EDTA, NaN_3_, and glucose-6-phosphate were purchased from Sigma-Aldrich Chemical Co. (St Louis, MO, USA). The solvents used in this study including ethanol, methanol, dichloromethane, ethyl acetate, petroleum ether, hexane, were of analytical reagent grade. The water used for all experiments was of distilled grade. *Candida albicans* strain ATCC 10231 was a kind gift from Dr. K. Marobela (Department of Biological Sciences, University of Botswana).

### Plant collection

*Combretum zeyheri* leaves were collected from Norton (Geographic coordinates of Norton, Zimbabwe, Latitude: 17°52’59”S, Longitude: 30°42’00”E, Elevation above sea level: 1360 m, Mashonaland West province of Zimbabwe). The plant was classified by Mr. Christopher Chapano, a taxonomist at the National Botanic Gardens (Harare, Zimbabwe). Herbarium samples (Voucher number N6E7) were kept at the Department of Biochemistry, University of Zimbabwe.

### Extraction

The preparation of plant extracts was previously described [11]. Briefly, 2 kg of *C. zeyheri* leaves were ground in a two speed blender (Cole Parmer Instrument Co., Vernon Hills, USA) and extracted with 8 L of absolute ethanol at room temperature. Extract was filtered through fine cloth and filtrate was decanted into preweighed labeled container. The solvent was removed under a stream of air in a fume cupboard at room temperature. The amount of solid extract was weighed and recorded.

### Isolation of active compounds from *Combretum zeyheri*

The number of compounds present in *C. zeyheri* leaf extract was determined by thin layer chromatography (TLC). Briefly, a small amount of *C. zeyheri* plant extract was dissolved in dichloromethane and was deposited as a spot on the TLC plate (aluminium-backed silica gel 60 F254, Sigma Aldrich, St Lous, MO, USA). The bottom edge of the plate was placed in a solvent reservoir (1 % methanol in dichloromethane), and the solvent moved up the plate by capillary action. When the solvent front reached the other edge of the stationary phase, the plate was removed from the solvent reservoir. The separated spots were visualized with ultraviolet lamp at 366 nm and by placing the plate in iodine vapor. Silica gel column chromatography was then used to purify individual chemical compounds from mixtures of compounds. For the assay, a slurry was prepared of the eluent (70 % dichloromethane in hexane) with the stationary phase powder (500 g of silica gel 60–120 mesh, Sigma Aldrich, St Louis, MO, USA) and then carefully poured into the column (8 cm internal diameter × 120 cm length). The column was left overnight to fully pack. The leaf extract of *C. zeyheri* (50 g) was adsorbed on 55 g of silica gel and loaded into the column. This layer was topped with cotton wool to protect the shape of the organic layer from the velocity of solvents. Different solvents (70 % dichloromethane in hexane, 80 % dichloromethane in hexane, 90 % dichloromethane in hexane, 100 % dichloromethane, 1 % methanol in dichloromethane, 2 % methanol in dichloromethane, 3 % methanol in dichloromethane, 5 % methanol in dichloromethane) were slowly passed through the column in order of their increasing polarity, to advance the organic material. Fractions of 500 ml were collected, vaporized and compounds present were checked by TLC. Samples which showed similar TLC profile were combined and further separation carried out using small columns (2.5 cm internal diameter × 60 cm length). Each solvent of 500 ml of 50 % dichloromethane in hexane, 60 % dichloromethane in hexane, 70 % dichloromethane in hexane, 80 % dichloromethane in hexane, 90 % dichloromethane in hexane and 100 dichloromethane was slowly passed through the column as before to advance the organic compounds in order of their increasing polarity. Fractions of 40 ml were collected, vaporized and compounds present were checked by TLC. Fractions which showed similar TLC profile were combined and further separation was carried out. For the fractions that showed presence of 3 or 2 compounds, crystallization was carried out by adding a few drops of hexane. The crystals formed were separated by filtration and their purity determined by TLC. Elucidation and characterization of the structures of the isolated compounds was carried out using ^1^H, ^13^C NMR techniques and UV spectroscopy.

### Antifungal activity determination of the isolated compounds

The antifungal activity of the *C. zeyheri* isolated compounds was investigated using the 3-(4,5-dimethylthiazol-2-yl)-2,5-diphenyltetrazolium bromide (MTT) assay, as described before [12]. Cultures of *Candida* species were transferred into fresh nutrient broth and 100 μl of fresh culture were added to each well of a 96-well microtitre plates. The *C. zeyheri* isolated compounds were dissolved in DMSO and added to each well to a final concentration of 45 μg/ml. Miconazole was used as the positive control and appropriate solvents used for dissolving the compounds were included as negative controls. Micro plates were incubated for 24 hours at 37 °C and 100 % relative humidity. As an indicator of growth 25 μl of 2 mg/ml MTT was added to each of the microtitre plate wells and incubated for three hours.

### Determination of the effect of combining active compound with miconazole

The antifungal activity of the isolated compound in combination with miconazole was assessed in a suspension assay by the checkerboard method. In brief, different concentrations of test compound were prepared by serial dilution and 20 μl of each concentration was added to the rows of a 96-well microtitre plate in diminishing concentrations. The final concentrations of the test compound in a microtitre plate ranged from 0–90 μg/ml. Miconazole was also added to the columns to final concentrations of 0–350 μg/ml. A 160 μl suspension of *Candida* strain adjusted to 1 × 10^6^ cfu/ml was added to each well and cultured at 37 °C for 24 hrs. The MIC of the test compound in combination with miconazole was determined using MTT as described before.

### Time-kill assays

The antifungal activities of miconazole and the isolated active compound alone and in combination against *C. albicans* were determined at 0. 4, 8, 20 and 24 hrs of incubation at 37 °C by colony-counting method. *Candida albicans* was exposed over time to active compound and miconazole alone as well as to their combinations. Test solutions were placed on a shaker and incubated at 37 °C. At the predetermined time point, an aliquot of 20 μl volumes were removed from each test suspension and plated on SDA for determining the colony forming units. Plates were then incubated at 37 °C for 24 h in an incubator (Jeio tech, South Korea) and viable colony counts were performed. The broth (SDB) without any agent was used as the control for *C. albicans* growth at each time point.

### Determination of the effect of active compounds on drug efflux pumps

Drug accumulation assays were carried out as described previously [12], using ciprofloxacin as the standard drug, to determine the effect of the isolated active compounds on ABC - drug efflux pumps in *C. albicans*. Further work was done to determine the concentration of compound that inhibits the activity of drug efflux pumps in *C. albicans* by half (IC_50_). IC_50_ measures the effectiveness of compound as drug efflux pump inhibitor therefore, different concentrations of compound (0–0.07) mg/ml were incorporated and the assay procedure was carried out.

### Determination of effect of active compound on ergosterol biosynthesis pathway

The effect of *C. zeyheri* plant compound on ergosterol biosynthesis in *C. albicans* was investigated by quantifying the amount of ergosterol produced by *C. albicans* in the presence and absence of the test compound at time intervals *in vitro*. Briefly, a single *C. albicans* colony from an overnight Sabouraud dextrose agar plate culture was used to inoculate each of the two flasks containing 400 ml of Sabouraud dextrose broth. The cells were grown until an OD of 0.5 was reached then *C. zeyheri* compound was added to one flask. The cultures were incubated at 37 °C and then 100 ml aliquots were withdrawn from each flask after 16 hrs and 24 hrs. The cells were harvested by centrifugation at 1077 × g-force for 5 min and washed once with sterile distilled water. Ten millilitres of 25 % alcoholic potassium hydroxide solution (25 g of KOH and 35 ml of sterile water, brought to 100 ml with 100 % ethanol) were added to each pellet and vortex mixed for 1 min. Cell suspensions were transferred to sterile tubes and incubated in an 85 °C water bath for 1 h. Following incubation, tubes were allowed to cool to room temperature. Sterols were extracted by adding a mixture of 4 ml sterile water and 10 ml n-hexane followed by vigorous vortex mixing for 3 min. The hexane layer was transferred to a clean tube and ergosterol content quantified spectrophotometrically at 293 nm using Unico UV-2800 spectrophotometer (UNICO United Products and Instruments Inc, Dayton, United States). The standard curve was used estimate the concentration of ergosterol produced in the absence and presence of *C. zeyheri* plant compound. For standards preparations, briefly, a stock solution of ergosterol (0.667 mg/ml) was diluted with ethanol to obtain concentrations of 0.01, 0.02, 0.03, 0.04 and 0.05 mg/ml and absorbances read spectrophotometrically.

### Determination of effect of active compound on *C. albicans* antioxidant enzymes

*Candida albicans* cells grown overnight at 37 °C in the presence of isolated antifungal active compound (50 μg/ml) were harvested by centrifugation and then suspended in homogenizing buffer (1 mmol/L phenylmethylsulphonyle fluoride, 250 mmol/L sucrose, 10 mmol/L Tris–HCl, pH 7.5). The control sample was also prepared and did not contain the active compound. The cells were then mechanically disrupted at 4 °C using a soniprobe (Polytron PT-MR 3000, Kinematica AG, Littau, Switzerland). The homogenate was collected and centrifuged at 20217 × g-force for 1 hour at 4 °C. The supernatant was collected and investigated for its protein concentration and antioxidant enzyme activity.

### Protein determination

The concentration of the antioxidant enzymes produced in the presence of *C. zeyheri* antifungal active compound was determined by the Lowry method [15]. A standard curve was generated from the concentrations of bovine serum albumin (BSA) standards and used to find the concentrations of the samples from their absorbances.

### Glutathione-S-transferases

GST activity was determined spectrophotometrically by measuring the formation of glutathione (GSH) and 1-chloro-2,4-dinitrobenzene (CDNB) conjugate at 340 nm [16]. One unit of GST activity is defined as the amount of enzyme producing 1 μmol of GS-DNB conjugate/min under the conditions of the assay. In this experiment, the assay mixture was placed in a 1 mL cuvette and consisted of potassium phosphate buffer pH 6.5 (875 μl), 50 μl of 20 mM glutathione, 50 μl of 20 mM 1-chloro- 2,4-dinitrobenzene (dissolved in ethanol), and 25 and 100 μl of supernatant. The reference cuvette contained everything except the supernatant being assayed. Results were expressed as nanomoles CDNB conjugate formed per minute per milligram protein by using a molar extinction coefficient of 9.6 × 10^3^ mol.L^−1^.cm ^−1^.

### Catalases

Catalase activity was investigated by measuring the decrease in absorbance of H_2_O_2_ at 240 nm due to hydrogen peroxide decomposition [17]. Potassium phosphate buffer pH 7.0 (1960 μl), 990 μl of 20 mM H_2_O_2_, 50 μl of supernatant were added to a quartz cuvette. Catalase activity was calculated in terms of nanomoles of H_2_O_2_ consumed per minute per milligram protein using the extinction coefficient 0.081 × 10^−1^ mol.L^−1^.cm^−1^. One unit was defined as 1 μM H_2_O_2_ reduced per min.

### Superoxide dismutase

Superoxide dismutase activity was determined using the adrenochrome assay which is based on the ability of SOD to inhibit the autoxidation of epinephrine in alkaline [18]. Briefly, during the reaction, SOD reacts with the O^2−^ formed during the epinephrine oxidation and therefore slows down the rate of formation of the adrenochrome as well as the amount that is formed. Because of this slowing process, SOD is said to inhibit the oxidation of epinephrine and a unit of SOD activity is defined as that amount of SOD required to cause 50 % inhibition of the oxidation of the epinephrine (SOD_50_). Activity was calculated by measuring the increase in absorbance of the solution containing 50 mM sodium carbonate buffer pH 10.2 (2685 μl), 75 μl of the supernatant, 200 μM adrenochrome (150 μl) and 90 μl of 0.01 M epinephrine at 480 nm.

### Glutathione reductase

Glutathione reductase activity was determined by the method in which 1 unit of GR activity is defined as the amount of the enzyme catalyzing the reduction of 1 μM of NADPH per min [19]. The reaction mixture contained 1.63 ml phosphate buffer pH 7.4, 0.025 ml supernatant, 0.1 ml NADPH, 0.1 ml EDTA and 0.1 ml oxidized GSH disulphide. Enzymatic activity was calculated by measuring the disappearance of NADPH at 340 nm and the results were expressed as nanomoles of NADPH oxidized per minute per milligram protein.

### Glutathione peroxidase

The activity of glutathione peroxidase was determined by measuring a decrease in absorbance at 340 nm, suggestive of the disappearance of NADPH [20]. The reaction mixture consisted of 1.53 mL phosphate buffer (0.05 mol/L, pH 7.0), 0.1 mL 1 mmol/L EDTA, 0.1 mL 1 mmol/L NaN_3_, 0.1 mL 1 mmol/L glutathione (GSH), 0.1 mL 0.2 mmol/L NADPH, 0.01 mL 0.25 mmol/L H_2_O_2_, and 100 μL supernatant in a final volume of 2.0 mL. The reaction was initiated by the addition of hydrogen peroxide and the enzyme activity was calculated as nanomoles of NADPH oxidized per minute per milligram protein by using a molar extinction coefficient of 6.22 × 10^3^ mol.L^−1^.cm^−1^.

### Glucose-6-phosphate dehydrogenase

Activity of Glucose-6-phosphate dehydrogenase was determined by measuring the reduction of NADP at 340 nm [21]. The reaction mixture contained 0. 3 ml Tris-HCI buffer, 0.1 ml NADP, 0.1 ml glucose-6-phosphate, 0.1 ml MgCI_2,_ 0.02 ml supernatant and 2.38 ml of H_2_O.

### Statistical analysis

A comparison of the antifungal tests and efflux activity of the samples with the standard efflux inhibitor, reserpine was evaluated by applying one way ANOVA using Dunnet’s Multiple Comparison Test as the post test which compares the difference between all samples versus the control sample. All values are expressed as the mean ± standard deviation and *P* < 0.05 values or less were considered to indicate statistically significant differences. Numerical data were analysed using Graphpad ™ version 5 for Windows, (Graphpad ™ Software Inc., San Diego, California, USA).

## Results

### Compounds isolated from *Combretum zeyheri*

**Fig. 1:**
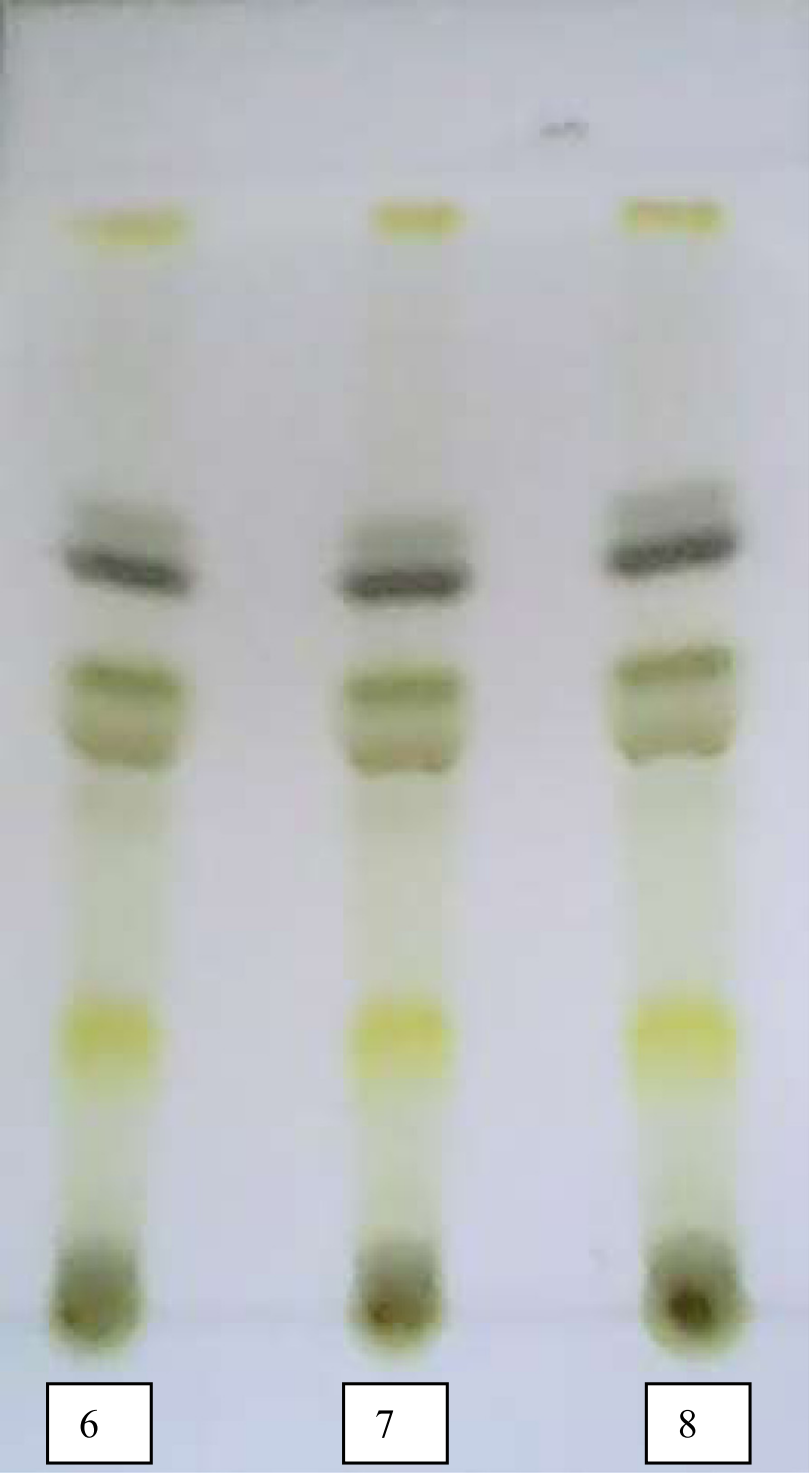
Typical TLC profile showing different *C. zeyheri* isolated fractions with similar compounds. Samples 6, 7 and 8 are different fractions separated by silica gel column chromatography using 90 % dichloromethane in hexane. The TLC plate was then run using ethyl acetate : hexane (1:3) as the mobile phase

**Fig. 2:**
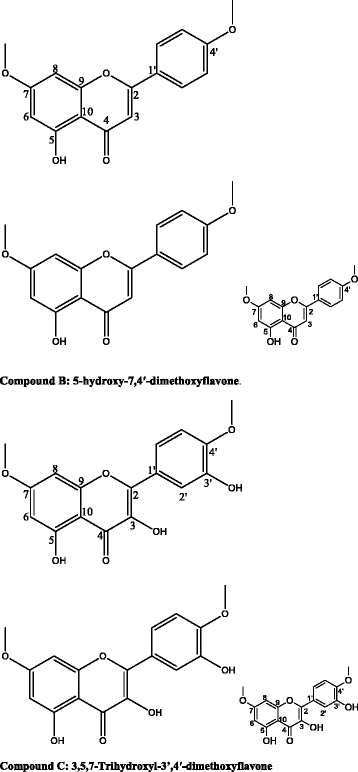
Flavonoids isolated from *Combretum zeyheri* from Norton, Mashonaland West, Zimbabwe. The flavonoids were characterized as 5-hydroxy-7,4’-dimethoxyflavone and 3,5,7-Trihydroxyl-3’,4’-dimethoxyflavone. These compounds have been isolated before from other plant species [23–25]

^**13**^**C NMR for compound B (CDCl**_**3**_**, 100 MHz)**: 163.9 (C2), 104.2 (C3), 182.4 (C4), 162.0 (C5), 98.0 (C6), 165.3 (C7), 92.5 (C8), 157.6 (C9), 105.4 (C10), 123.4 (C1’), 127.9 (C2’), 114.4 (C3’), 162.5 (C4’), 114.4(C5’), 127.9 (C6’), 55.8 (C6’– OCH_3_ ), 55.5(C7 – OCH_3_).

^**1**^**H NMR data for 3,5,7-trihydroxyl-3’,4’-dimethoxyflavone, or compound C (MeOD, 400 MHz) was as follows**: 12.5 (1H, s,); 7.76 (1H, *br*s); 7.73 (1H, d, 8Hz); 6.92 (1H, d, 8 Hz); 6.76 (1H, *br*s); 6.33 (1H, d, 4Hz); 3.84 (3H, s); 3.84 (3H, s).

^**13**^**C NMR for compound C (MeOD, 100 MHz)**: 147.4 (C2), 136.6 (C3), 176.4 (C4), 160.7 (C5), 97.9 (C6), 166.3 (C7), 92.5 (C8), 156.5 (C9), 104.4 (C10), 122.3 (C1’), 115.9 (C2’), 147.8 (C3’), 149.4 (C4’), 112.0 (C5’), 122.3 (C6’), 56.5 (C6’– OCH_3_ ), 56.2 (C7 – OCH_3_).

Separation of *C. zeyheri* plant compounds resulted in 97 different fractions which were then combined according to their TLC profile similarities (Fig. 1). Further separation by silica gel column chromatography resulted in fractions which had 3 or 2 different compounds, according to TLC profiles. Compounds were then crystallized by adding hexane and then filtered through Whatman paper. Yellow, amorphous solid crystals were observed in four fractions so they were filtered and purity of the crystals checked by TLC. Thus, four pure compounds were isolated from *C. zeyheri* and were labeled A-D. The structures of compounds B and C were elucidated using spectroscopic techniques and were characterized as 5-hydroxy-7,4’-dimethoxyflavone and 3,5,7-trihydroxyl-3’,4’-dimethoxyflavone respectively (Fig. 2). ^**1**^**H NMR data for 5-hydroxy-7,4’-dimethoxyflavone, or compound B (CDCl**_**3**_**, 400 MHz) was as follows**: 12.8 (1H, s,); 7.78 (2H, dd, 8Hz, 4Hz, H2’ and H6’); 6.97 (2H, dd, 8Hz, 4 Hz, H3’and H5’); 6.52 (1H, s, H3); 6.43 (1H, d, 4Hz, H8); 6.32 (1H, d, 4Hz, H6); 3.86(3H, s); 3.85 (3H, s).

### Antifungal activity determination of the *Combretum zeyheri* isolated compounds

**Fig. 3:**
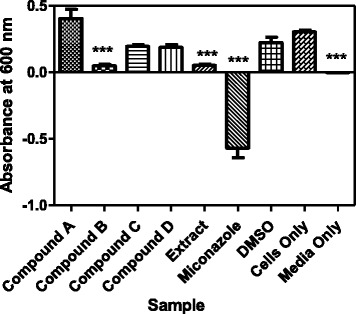
Effect of *C. zeyheri* compounds on growth of *C. albicans*. Generally a decrease in growth of cells was observed in the presence of Compound B, *C. zeyheri* extract and the standard inhibitor miconazole. The results are comparable to media only, where no cells were added. Values are mean ± SD for *N* = 3 and *P < 0.0001

The four compounds that were isolated from *C. zeyheri* were tested for their antifungal activity and the results are shown in Fig. 3. Compound 5-hydroxy-7,4’-dimethoxyflavone showed antifungal activity against *C. albicans* using the broth dilution method [10]. This is shown by a decrease in growth of the cells as indicated by a decrease in absorbance when compared to the control which contained cells only.

### Effect of combining 5-hydroxy-7, 4’-dimethoxyflavone with miconazole

**Fig. 4:**
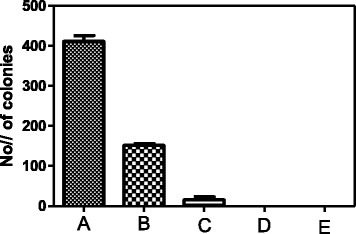
Synergistic activity of 5-hydroxy-7,4’-dimethoxyflavone-miconazole mixture against *C. albicans*. (A – 22. 5 μg/ml compound B, B – 90 μg/ml compound B, C – 150 μg/ml Miconazole, D – 150 μg/ml Miconazole + 22.5 μg/ml compound B, E - 150 μg/ml Miconazole + 90 μg/ml compound B.) Values are mean ± SD for *N* = 3. Complete inhibition of growth of *C. albicans* was observed when 5-hydroxy-7,4’-dimethoxyflavone and miconazole were combined, unlike when the two were tested on their own

The *in vitro* combined antifungal activity of 5-hydroxy-7, 4’-dimethoxyflavone with miconazole against *Candida albicans* was assessed. Results show that 5-hydroxy-7, 4’-dimethoxyflavone in combination with miconazole exhibited severe growth inhibition than miconazole alone or 5-hydroxy-7, 4’-dimethoxyflavone alone, at all the concentrations tested (Fig. 4).

### Time-kill assays

**Fig. 5:**
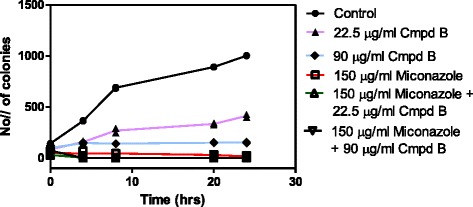
Representative time-kill curves of *Candida albicans* following exposure to miconazole, Compond B (5-hydroxy-7,4’-dimethoxyflavone) and miconazole combined with Compound B. Control represents the untreated *Candida* cells. There was decrease on the rate at which the *Candida* cells treated with 5-hydroxy-7,4’-dimethoxyflavone-Miconazole mixture were growing as compared to the untreated cells and those treated with miconazole alone or 5-hydroxy-7,4’-dimethoxyflavone alone

Synergism was further confirmed with time-kill curves (Fig. 5). Fungistatic effect on the growth of *Candida* cells was observed for both miconazole and 5-hydroxy-7, 4’-dimethoxyflavone at 150 μg/ml and 22.5 μg/ml respectively. The fungistatic activity of these two was transformed to fungicidal by combining them. After only 4 h of incubation, the combination yielded a decrease to zero colonies in comparison to miconazole alone.

### Effect of 5-hydroxy-7, 4’-dimethoxyflavone on *Candida albicans* drug efflux pumps

**Fig. 6:**
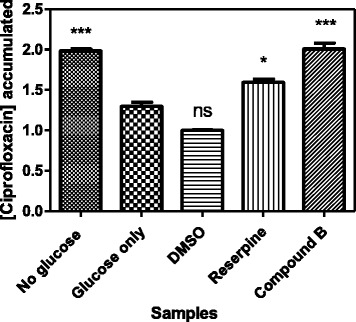
Graph showing the concentrations of ciprofloxacin that accumulated in *C. albicans* in the presence of glucose only, DMSO, reserpine and compound B. Values are mean ± SD for *N* = 3 and *P < 0.05. Cells which were exposed to glucose had the least amount of the ciprofloxacin left in the cells, thus, indicating active efflux with glucose serving as a source of energy. Cells exposed to glucose and 5-hydroxy-7,4’-dimethoxyflavone had a higher amount of dye, thus, indicating the inhibition of the efflux of the ciprofloxacin from the cells. Reserpine, a standard inhibitor efflux inhibitor showed the inhibition of drug efflux and DMSO was the negative control and solvent used to dissolve the compound 5-hydroxy-7, 4’-dimethoxyflavone

**Fig. 7:**
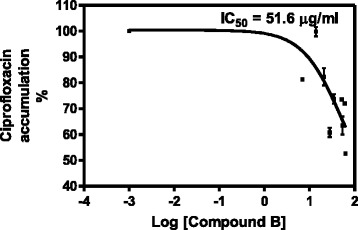
Determination of IC_50_ for Compound B on *C. albicans’* efflux pumps inhibitory activity. Values are mean ± SD for *N* = 3. The IC_50_ for Compound B was found to be 51.64 μg/ml

The compound, 5-hydroxy-7, 4’-dimethoxyflavone was investigated for its effect on ABC drug transporters in *Candida albicans* by measuring the concentration of ciprofloxacin that accumulated in *C. albicans* in its presence (Fig. 6). Glucose was added to provide energy for the drug efflux pumps, therefore, cells which were exposed to glucose only had the least amount of the ciprofloxacin that accumulated in cells since the ciprofloxacin was pumped out of the cells. Cells exposed to 5-hydroxy-7, 4’-dimethoxyflavone retained a higher amount of ciprofloxacin, thus, indicating the inhibition of the efflux of the drug from the cells. Reserpine, a standard inhibitor efflux inhibitor showed the inhibition of drug efflux as expected. Thus, further work was carried out to determine the concentration of 5-hydroxy-7, 4’-dimethoxyflavone that reduced the percentage activity of the drug efflux pumps by half, IC_50_ determination. The IC_50_ for 5-hydroxy-7, 4’-dimethoxyflavone was found to be 51.64 μg/ml (Fig. 7).

### Effects of 5-hydroxy-7, 4’-dimethoxyflavone on ergosterol biosynthesis pathway

**Fig. 8:**
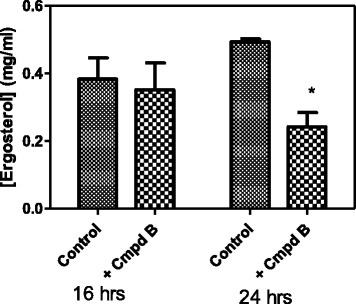
Effect of production of ergosterol by *C. albicans* in the presence and absence of Compound B at times 16 and 24 hours. Values are mean ± SD for *N* = 3 and *P < 0.05. A decrease in ergosterol produced by *C. albicans* in the presence of 5-hydroxy-7,4’-dimethoxyflavone was observed with time

Sterol assay was performed for the mechanistic analysis of the tested drug. Most antifungal drugs target the ergosterol biosynthesis pathway, particularly the enzyme lanosterol 14α-demethylase, and disrupt membrane fluidity, asymmetry and integrity. The effects of 5-hydroxy-7, 4’-dimethoxyflavone on lanosterol 14α-demethylase, was investigated. Figure 8 shows total sterol content of *C. albicans* treated with ½ MIC of 5-hydroxy-7, 4’-dimethoxyflavone at times 16 and 24 hrs of cell incubation. The compound 5-hydroxy-7, 4’-dimethoxyflavone showed ergosterol inhibition in *C. albicans* to 91. 6 and 63 % at 16 and 24 hrs respectively, at the tested concentration (Table 1). The decrease in ergosterol in *C. albicans* could be due to slight inhibition of the ergosterol biosynthetic pathway. Results from Table 1 show a time-dependent decrease in ergosterol synthesis as a result of the effect of 5-hydroxy-7, 4’-dimethoxyflavone.

**Table 1:** Time-dependent effect of 5-hydroxy-7,4’-dimethoxyflavone on ergosterol production by *C. albicans*

Time of incubation (hrs)	[Ergosterol] % decrease
16	91.6
24	63.0

### Effects of 5-hydroxy-7, 4’-dimethoxyflavone on antioxidant enzymes

*Candida albicans* has evolved enzymatic antioxidant defense mechanisms in order to minimise the damaging effects of ROS produced by phagocytes during an infection, thus 5-hydroxy-7, 4’-dimethoxyflavone was investigated for its effect on these antioxidant enzymes. The activities of the antioxidant enzymes SOD, CAT, GST, GPx, GR and G6PD were investigated spectrophotometrically by measuring change in absorbance of samples in the presence or absence of the compound 5-hydroxy-7,4’-dimethoxyflavone. Specific activities of antioxidant enzymes were then calculated using the extinction coefficients provided for each enzyme and results recorded in Table 2. Results show that 5 μM of 5-hydroxy-7,4’-dimethoxyflavone resulted in complete inhibition of activity of the enzymes superoxide dismutase, glutathione peroxidase, glutathione reductase, glutathione-S-transferase and glucose-6-phosphate dehydrogenase. However, treating C*andida albicans* with 5-hydroxy-7,4’-dimethoxyflavone resulted in roughly a 26 % decrease in activity of the enzyme catalase.

**Table 2:** Specific activities of antioxidant enzymes

Enzyme	Substrate	Specific activity (U/mg protein)	
		No compound	+ Compound
Glutathone-S-transferase	CDNB	0.253 ± 0.015	_
Catalase	H_2_O_2_	1.396 ± 0.068	1.031 ± 0.064
Superoxide dismutase	Epinephrine	0.204 ± 0.05	_
Glucose 6 phosphate dehydrogenase	NADP	387.9 ± 26	_
Glutathione reductase	NADPH	39.77 ± 4.8	_
Glutathione peroxidase	NADPH	0.00022	_

*N* = 3

## Discussion

Plants have been reported as a valuable source of new natural products despite the availability of different approaches for the discovery of therapeutics. Studies involving the isolation, characterization and purification of the chemical compounds of the plant and screening for antifungal activity have been reported to be more than promising [22]. The assumption for these studies was that they may result in the development of a potent entity which will be of lower toxicity and a high therapeutic value to mankind. In this current study, the air-dried leaves of *C. zeyheri* were extracted successively with ethanol followed by various chromatographic techniques using different solvents. Subsequent chromatographic assays on silica gel yielded compounds 5-hydroxy-7, 4’-dimethoxyflavone and 3, 5, 7-trihydroxyl-3’, 4’-dimethoxyflavone (Fig. 2). The compounds were investigated for their antifungal activity and 5-hydroxy-7, 4’-dimethoxyflavone was found to have an MIC of 45 μg/ml against *C. albicans* (Fig. 3). This is the first time that the antifungal activity of 5-hydroxy-7, 4’-dimethoxyflavone against *C. albicans* has been reported using the broth dilution assay. The compound 5-hydroxy-7,4’-dimethoxyflavone has already been discovered in the methanol extract of aerial parts of *Salvia poculata* Nab., a Turkish endemic *Salvia* species [23] and had showed antioxidant activity using *β*-carotene bleaching, and superoxide anion radical and 2,2’-azino-bis(3-ethylbenzothiazoline-6-sulphonic acid (ABTS) cation radical scavenging activity assays. The compound 5-hydroxy-7, 4’-dimethoxyflavone has also been isolated from *Combretum erythrophylum* and was found to have antimicrobial activity against *Vibrio cholerae* and *Enterococcus faecalis*, with MIC values ranging from 25–50 μg/ml [24]. However, the same study found it to be toxic to human cells and to have the poorest antioxidant activity. In another study, 5-hydroxy-7, 4’-dimethoxyflavone was isolated from the rhizome of *Kaempferia parviflora,*which has been used for the treatment of allergy and gastrointestinal disorders as well as an aphrodisiac and for fungal infections [25]. However, this compound was found to have no antifungal activity against the dermatophytes *Trichophyton rubrum, Trichophyton mentagrophytes and Microsporum gypseum* at the highest concentration of 250 μg/ml tested. The antifungal activity of this compound was not investigated against *C. albicans* in this same study. Therefore, this current study reports the successful isolation of 5-hydroxy-7, 4’-dimethoxyflavone from *C. zeyheri* leaf extract, which we previously reported to have antifungal activity [11].

Further work was carried out to determine the combined activity of 5-hydroxy-7, 4’-dimethoxyflavone with miconazole since synergism has become a possible thrust area in developing therapeutic strategies against infectious diseases. The results demonstrated that 5-hydroxy-7,4’-dimethoxyflavone is very effective as antifungal agent, as it inhibited *C. albicans* at 22.5 μg/ml and showed synergy with miconazole as shown by a decrease in growth of *C. albicans* cells in the presence of 5-hydroxy-7,4’-dimethoxyflavone-miconazole mixture as compared to 5-hydroxy-7,4’-dimethoxyflavone alone or miconazole alone (Figs. 4 and 5). Thus, the efficacy of antifungal agents can be improved by using combination therapy where these drugs are administrated along with other chemosensitizing agents.

The isolated 5-hydroxy-7, 4’-dimethoxyflavone was further investigated for its inhibitory activity on ABC drug efflux pumps in *C. albicans* by monitoring an increase in ciprofloxacin, assessing the level of its accumulation, in response to reserpine. There was a higher accumulation of ciprofloxacin in *Candida* cells in the presence of 5-hydroxy-7, 4’-dimethoxyflavone than with reserpine (Fig. 6). The compound 5-hydroxy-7, 4’-dimethoxyflavone demonstrated the activity in a dose-dependent manner with IC_50_ value of 51.64 μg/ml (Fig. 7). These results support those obtained from synergism assays where by the underlying synergistic antifungal mechanisms could be due to blockage of ABC efflux pumps and increasing the susceptibility of *Candida* to miconazole. Further work would be necessary to establish the major mechanism which could be competitive inhibition of drug efflux pumps or non competitive inhibition. It also would be of great interest to determine whether or not 5-hydroxy-7, 4’-dimethoxyflavone can inhibit the activity of other *Candida* drug transporters such as CaMdr1p belonging to major facilitator superfamily. The study of inhibitors of the drug efflux pumps in *Candida* species is of great importance but to date only limited in vivo testing has been done and no human toxicology of promising candidates has been performed [26]. The challenges that have so far been reported include that the search for candidate efflux pump inhibitors can be costly and time consuming since substantial efforts are needed before inhibitors of efflux pumps can be used clinically and be fully accepted by the medical community.

This study also focused on investigating the effect of the 5-hydroxy-7, 4’-dimethoxyflavone on the *C. albicans* ergosterol biosynthesis. Ergosterol biosynthesis is an important target for the development of novel antimycotic drugs, and that of *C. albicans* has been proposed with 14 sterol intermediates resulting in ergosterol and another secondary final compound C-24 ethylsterol [27]. Classes of antimycotics that are curently used to target the ergosterol biosynthesis pathway and disrupt membrane fluidity, asymmetry and integrity are the azoles, allylamines, polyenes, and morpholines [28]. Analysis of ergosterol obtained from tested *Candida albicans* showed a time-dependent decrease to 91 % and 63 % at 16 and 24 hrs respectively, in ergosterol content in the cells treated with ½ MIC of 5-hydroxy-7, 4’-dimethoxyflavone (Fig. 8 and Table 1). This study supports the work which previously showed that most of the ergosterol inhibitors were fungicidal in its action [29]. This could be due to direct damage to the cell membrane. However, the exact mode of action 5-hydroxy-7, 4’-dimethoxyflavone need to be elucidated. Further studies with a higher number of azole-resistant strains should help elucidate whether 5-hydroxy-7, 4’-dimethoxyflavone maintains a good level of activity in all cases. If this is the case, 5-hydroxy-7, 4’-dimethoxyflavone, alone or in combination with azoles, could represent an alternative for the treatment of *C. albicans* infections.

Antioxidant enzymes have a role in the protection of *C. albicans* against oxidative stress, thus, inhibition of these enzymes by 5-hydroxy-7, 4’-dimethoxyflavone may be a step toward the search for new targets for antifungal agents. The results obtained in this study showed complete inhibition of the activity for all the enzymes tested except for catalase which showed a decreased activity in the presence of 5-hydroxy-7, 4’-dimethoxyflavone (Table 2). However, observed decrease in specific activity or complete inhibition of activity may not be due to direct effects of the compound on enzymes because previously, 5-hydroxy-7,4’-dimethoxyflavone has been reported to have an antioxidant activity, thus, may have quenched the reactive oxygen species before *C. albicans* produced antioxidant enzymes [23].

## Conclusion

The compound responsible for the antifungal activity of the leaf extract of *Combretum zeyheri* was successfully isolated and characterized as 5-hydroxy-7, 4’-dimethoxyflavone.

The mechanism of action of 5-hydroxy-7, 4’-dimethoxyflavone as an antifungal includes inhibition of *C. albicans*’ ergosterol synthesis, drug efflux pumps as well as the antioxidant enzymes.

## Abbreviations

MTT: 3-(4,5-dimethylthiazol-2-yl)-2,5-diphenyltetrazolium bromide
SDA: Sabouraud dextrose agar
MIC: Minimum inhibitory concentration
TLC: Thin layer chromatography
ABC: ATP binding cassette
OD: Optical density
BSA: Bovine serum albumin
ATCC: American type culture cell
CDNB: 1-Chloro-2,4-dinitrobenzene
DMSO: Dimethyl sulfoxide
EDTA: Ethylenediaminetetraacetic acid
IC_50_: Half maximal inhibitory concentration
SOD: Superoxide dismutase
GST: Glutathione-s-transferase
CAT: Catalase
GR: Glutathione reductase
G6PD: Glucose 6 phosphate dehydrogenase
GPx: Glutathione peroxidase
GSH: Glutathione
NADP: Nicotinamide adenine dinucleotide phosphate
H_2_O_2_: Hydrogen peroxide
